# Effect of flaxseed on systemic inflammation and oxidative stress in diabetic rats with or without chronic kidney disease

**DOI:** 10.1371/journal.pone.0258800

**Published:** 2021-10-19

**Authors:** Mohammed Al Za’abi, Haytham Ali, Badreldin H. Ali

**Affiliations:** 1 Department of Pharmacology and Clinical Pharmacy, College of Medicine and Health Sciences, Sultan Qaboos University, Muscat, Oman; 2 Department of Animal and Veterinary Sciences, College of Agricultural and Marine Sciences, Sultan Qaboos University, Muscat, Oman; Universidade Federal do Rio de Janeiro, BRAZIL

## Abstract

**Background:**

Diabetes mellitus (DM) and chronic kidney disease (CKD) are common causes of morbidity and mortality. Flaxseed contains several bioactive compounds that have been shown to possess anti-inflammatory and antioxidative properties. The aim of the present study was to investigate the possible effect of flaxseed in diabetic rats with adenine–induced CKD.

**Methods:**

Male Wister rats (n = 48) were randomly divided into seven equal groups and treated for 33 consecutive days as follows: G1: control. G2 adenine, G3: streptozotocin (STZ), G4: flaxseed, G5: adenine+flaxseed, G6: STZ+flaxseed, G7: adenine+STZ+flaxseed). DM or CKD were experimentally induced by a single intraperitoneal injection of streptozotocin (STZ) or by adenine via oral gavage, respectively.

**Results:**

Rats fed adenine alone exhibited several changes including decreased body weight, increased food and water intake and urine output, increased urinary albumin/creatinine ratio. They also showed an increase in plasma urea and, creatinine, indoxyl sulfate, neutrophil gelatinase-associated lipocalin and cystatin C, and a decrease in renalase activity. These were associated with significant changes in inflammatory and oxidative biomarkers, e.g., increase in 8-isoprostane, 8 -hydroxy -2-deoxy guanosine and decrease in antioxidant enzymes, as well as increase in interleukins 1β and 6, and NF-κB, and a decrease in interlukin-10. Histopathologically, there was increased tubular necrosis and fibrosis. Concomitant administration of adenine and STZ further worsened the renal damage induced by adenine alone. Flaxseed significantly ameliorated the changes caused by adenine and STZ, given either singly or in combination.

**Conclusion:**

These findings suggest that flaxseed is a potential therapeutic agent in attenuating the progression of CKD in diabetes.

## Introduction

Chronic kidney disease (CKD) is a significant cause of morbidity and mortality [1]. The global prevalence of CKD in 2017 was estimated to be 9.1%, which is 29.3% higher than what it was in 1990 [2, 3], which has led to a significant burden on the health resources and society as CKD patients are at increased risks of other diseases such as hypertension, stroke, anaemia, osteoporosis and depression [4].

Diabetes mellitus (DM) is among the most common chronic non-communicable diseases globally, and is considered a serious public health concern [5]. The World Health Organization (WHO) indicates that the prevalence of diabetes globally, and especially in Arabian Gulf countries, is very high, imposing a steady increase in health expenditure [6]. DM is a progressive disease that can lead to multiple complications, including nephropathy, neuropathy, retinopathy, and others [7]. It is one of the major risk factors for the development of CKD. It is estimated that 38% of all causes of end-stage kidney diseases (ESKD) in the United States of America is attributed to diabetes [8]. Therefore, the identification and development of new therapeutic agents for CKD and diabetes are highly needed.

Inflammation and oxidative stress have been shown to play important roles in the pathogenesis and progression of both DM and CKD [9, 10]. Patients suffering from DM and/or CKD have a low grade but persistent inflammation, which contributes to their complications and mortality [11]. The concentrations of the inflammatory and oxidative biomarkers and mediators were found to be inversely related to the levels of renal function as well as diabetic micro- and macrovascular complications [11, 12]. Thus, agents that suppress inflammation and enhance antioxidative capacity may be considered as potential adjunctive therapy for patients with DM and CKD.

Flaxseed (*Linum usitatissimum*), has been found in folk medicine to be beneficial in the treatment of several diseases [13, 14]. It contains 32–45% of its mass as oil of which 51–55% is α-linolenic acid [13]. It also contains three different types of phenolic compounds–phenolic acids, flavonoids and lignans [15]. Some of these compounds e.g., secoisolariciresinol diglucoside (SDG) have been shown to possess antioxidant, hypolipidemic and hypoglycemic effects [16].

Flaxseed has been found to affect gut functions and lipid metabolism in rats and possess an antihypertensive effect via the modulation of endogenous enzymes in deoxycorticosterone acetate induced renal hypertension in rats [17, 18].

In the kidney, flaxseed oil reduced the renal injury in experimental polycystic kidney disease [19]. It also decreased the C-reactive protein and inflammation in chronic hemodialysis patients [20]. Furthermore, a mixture of flaxseed and pumpkin seeds exhibited significant hypoglycemic, hypolipidemic and nephroprotector effects in alloxan-induced diabetic rats [21].

In this study, we examined the effects of flaxseeds on experimentally induced diabetes in rats with or without experimentally induced CKD.

## Materials and methods

### Animals and experimental work

Male Wistar rats (9–10-week-old, weighing 240±10 g) were obtained from the Sultan Qaboos University (SQU) small animal house. They were housed in metabolic cages and maintained on a 12-hour light-dark cycle (lights on at 6:00 a.m.), an ambient temperature of 22±2°C and relative humidity of about 60%. Animals were supplied with additive-free and nutritionally- adequate pelleted food and water ad libitum (0.85% phosphorus, 1.12% calcium, 0.35% magnesium, 25.3% crude protein, and 2.5 IU/g vitamin D3, Oman Flour Mills, Muscat, Oman). Ethical approval was obtained from the Sultan Qaboos University Animal Ethics Committee before the start of the experimental work.

Following one week of acclimatization, rats (n = 48) were randomly divided into eight equal groups (n = 6 per group) and treated for 33 consecutive days. Flaxseed was mixed with the diet at a concentration of 15%^w/w^ as described before [22]. The details of the treatments of the different groups are summarized in Table 1.

**Table 1:** Experimental design.

Group number	Name (treatment)	Code	Adenine (150 mg/Kg/day orally for 20 days, from day 14–33)	Streptozotocin (55 mg/Kg as single intraperitoneal injection on day 11)	Flaxseed (15%^w/w^ orally in the feed for 33 days)
1	Control	CON	-	-	-
2	Adenine	ADE	+	-	-
3	Streptozotocin	STZ	-	+	-
4	Adenine + Streptozotocin	ADE+STZ	+	+	-
5	Flaxseed	FLX	-	-	+
6	Adenine + Flaxseed	ADE+FLX	+	-	+
7	Streptozotocin + Flaxseed	STZ+FLX	-	+	+
8	Adenine + Streptozotocin + Flaxseed	ADE+STZ+FLX	+	+	+

During the treatment period, the rats were weighed weekly, and a day before the last day of treatment, they were individually placed in metabolic cages to collect the urine voided in the last 24 h. At the end of the treatment, the rats were anesthetized with ketamine (75 mg/kg) and xylazine (5 mg/kg) intraperitoneally, and blood (about 5 mL) was collected from the anterior vena cava and placed into heparinized tubes and centrifuged at 900 g at 4°C for 15 min to separate plasma. The rats were then sacrificed by an overdose of anesthesia and the heart was excised to ensure that the rat does not recover. Th two kidneys were excised, blotted on filter paper and weighed. The right kidney and most of the left one were rapidly dipped in liquid nitrogen and kept frozen at -80° C for conducting biochemical tests. A small piece of the left kidney was placed in formol-saline for subsequent histopathological examination. Precautions were taken to minimize the suffering to the animals throughout the study and all procedures involving animals and their care were carried out per international laws and policies (EEC Council directives 2010/63/EU, 22 September 2010 and NIH Guide for the Care and Use of Laboratory Animals, NIH Publications, 8th edition, 2011).

### Induction of CKD and DM

CKD was induced by the administration of adenine (150 mg/Kg/day) via oral gavage as a suspension in 2%^w/v^ carboxymethylcellulose [23]. The control and other groups not treated with adenine received carboxymethylcellulose. Diabetes was induced by a single intraperitoneal injection of STZ (55 mg/Kg) dissolved in 0.1 M citrate buffer (pH 4.5). Other groups of rats were injected with normal saline. DM status was ascertained by measuring the fasting glucose concentration in the blood, obtained by pricking the tail tip, two days after STZ injection using a OneTouch® UltraMini® Meter (LifeScan, Milpitas, CA, USA) were a concentration of ≥ 14 mmol/L was considered establishment of DM.

### Chemicals and biochemical methods

Adenine and STZ were bought from Sigma (St. Louis, MA., USA). Flaxseed powder was purchased from Walmart Inc. (Bentonville, AR, USA). The rest of the chemicals and reagents used were of highest commercially available purity.

Urea, uric acid, albumin, glucose, calcium, creatinine and phosphorus were measured using an automated biochemical analyzer, Mindray BS-120 Chemistry Analyzer, from Shenzhen Mindray Bio-Medical Electronics Co, Ltd. (Shenzhen, P. R. China). Renalase, 8-hydroxy-2’-deoxyguanosine (8-OHdG), nuclear Factor kappa-light-chain-enhancer of activated B cells (NF-κB), N-Acetyl-β-D-Glucosaminidase (NAG) and advanced glycation end products (AGEs) were measured using ELISA kits from Cusabio Biotech Co. Ltd. (Wuhan, Hubei Province, P. R. China). Indoxyl sulfate and 8-isoprostane were measured using ELISA kits from MyBioSource Inc. (San Diego, CA, USA). Catalase (CAT), total antioxidant capacity (TAC) and superoxide dismutase (SOD) were estimated using colorimetric assay kits from BioVision (Milpitas, CA, USA). Cystatin C, interleukin-1β (IL-1β), interleukin-6 (IL-6) and neutrophil gelatinase-associated lipocalin (NGAL) were measured using ELISA kits from Thermo Fisher Scientific (Waltham, MA, USA). Interleukin-10 (IL-10) was estimated using an ELISA kit from Abcam (Cambridge, UK). Osmolality was measured by an osmometer (Osmomat 3000, Gonotec GmbH, Berlin, Germany) using the freezing point depression method.

### Histopathological methods

The kidneys were cut into 4 μm sections and stained with three stains: Hematoxylin and Eosin (H & E), Picro-Sirius red (ab150681, Abcam) and Periodic acid Schiff (PAS) (ab150680, Abcam) stains. The percentage of renal tubular necrosis was scored by semi-quantitative method on a scale 0–4 as following; 0 = normal, no necrosis; 1 <10%; 2 = 10–25%; 3 = 26–75%; 4 >75% as previously described [24, 25]. Three 40X fields were evaluated from each kidney section of each animal of the eight groups, and the mean percentage was converted to the score value. Fibrosis was assessed using the Picro-sirus red stain, and tubular atrophy was evaluated through the PAS stain.

Sirius red-stained slides were analyzed following the procedure described by Manni et al., 2011 [26]. The slides were examined by Olympus B51X microscope attached to Olympus DP70 camera and images were acquired using the x40 objective lens. Three random images of the renal cortex were acquired from each kidney of each animal of the 5 groups and stored as TIFF 24-bit RGB colour image files. All images were captured using the same camera and microscope settings. Image analysis was performed on the stored images using ImageJ® image analysis software (http://rsbweb.nih.gov/ij/). Briefly, the images were converted into greyscale, and the red-stained collagen was isolated using the hue histogram filter available in “Threshold Colour” followed by measuring the isolated area as a percentage. Fibrosis assessed the tissues’ collagen content by calculating the ratio of the mean Sirius red-stained positive area to the whole mean area of each section for each animal.

### Statistical analysis

Data were expressed as means ± SEM and were analyzed with GraphPad Prism version 5.03 for Windows software (GraphPad Software Inc., San Diego, CA, USA). Comparisons between the groups were performed by one-way analysis of variance (ANOVA), followed by Bonferroni comparisons. P values < 0.05 were considered significant.

## Results

### Physiological parameters

Adenine and STZ administered alone, or concomitantly induced significant changes in the measured physiological parameters when compared with control. They significantly reduced body weight and significantly increased relative kidney and liver weight, water intake and food intake and urine flow. Flaxseed, when combined with adenine, STZ or both, significantly abated most of these changes. However, it did not induce any significant changes in these parameters when given alone (Table 2).

**Table 2:** Effect of flaxseed (FLX) treatment on some physiological characteristics in control rats (CON), and rats administered concomitantly adenine (A) and /or streptozotocin (STZ).

Parameters/ Treatment	CON	ADE	STZ	ADE+STZ	FLX	ADE+FLX	STZ+FLX	ADE/STZ/FLX
Initial body weight (g)	238.17±18.63	240.33±18.89	241.17±9.01	240.5±19.31	240.67±6.85	241.67±21.37	240.33±8.16	243.5±13.45
Final body weight (g)	304.17±25.79	234.67±18.98^**a**^	195.83±8.28^**a**^	180.67±14.39^**a**^^**b**^	315.83±9.58	276.83±23.62	256.67±9.17^**c**^	230.5±15.15^**a**^^**d**^
Change in body weight (%)	27.36±2.58	-2.43±0.49^**a**^	-18.8±1.59^**a**^	-24.87±1.2^**a**^^**b**^^**c**^	31.27±2.21	14.68±0.83^**a**^^**b**^	6.77±0.63^**a**^^**c**^	-5.59±1.16^**a**^^**c**^^**d**^
Relative kidney weight (%)	0.6±0.02	1.24±0.08^**a**^	0.93±0.04^**a**^	1.28±0.07^**a**^^**c**^	0.59±0.02	0.94±0.04^**a**^^**b**^	0.88±0.04^**a**^	1.23±0.05^**a**^^**c**^
Relative liver weight (%)	1.07±0.05	1.15±0.04	1.31±0.09^**a**^	1.42±0.07^**a**^^**b**^	1.05±0.06	1.06±0.06	1.17±0.07	1.29±0.06^**a**^
Water intake (mL)	25.42±2.08	56.67±1.79^**a**^	171.67±4.59^**a**^	186.67±4.77^**a**^^**b**^^**c**^	27.5±2.42	43.75±2.21^**a**^^**b**^	122.5±6.68^**a**^^**c**^	134.17±5.69^**a**^^**b**^^**c**^^**d**^
Urine flow (μl/min)	7.7±0.77	25.81±1.01^**a**^	89.81±2.79^**a**^	96.64±4.21^**a**^^**b**^	8.8±0.98	19.68±1.62^**a**^	62.27±3.62^**a**^^**c**^	67.25±3.69^**a**^^**b**^^**c**^^**d**^
Food intake (g)	20.53±1.51	17.75±0.89	25.78±0.43	22.35±2.25	28.43±2.1^**a**^	21.52±1.58	36.97±1.69^**a**^^**c**^	35.1±3.13^**a**^^**b**^^**c**^^**d**^
Fecal output (g)	6.45±0.55	6.0±0.55	11.1±0.45^**a**^	11.05±1.0^**a**^^**b**^	9.72±0.8^**a**^	7.32±0.5	16.8±1.39^**a**^^**c**^	14.6±1.43^**a**^^**b**^^**c**^^**d**^

Values in the table are means ± SEM (n = 6).

Diabetes was induced by a single intraperitoneal injection of streptozotocin (55 mg/kg) and chronic kidney disease by adenine (150 mg/kg/day) by oral gavage and flaxseed (15% w//w) was given throughout the experiment by oral gavage.

Differences between the groups were assessed by one-way analysis of variance (ANOVA) followed by Bonferroni’s multiple comparison test, where P < 0.05.

^a^denotes significance of different groups vs. control group.

^b^denotes significance of different adenine treated groups vs. adenine alone group.

^c^denotes significance of different streptozotocin groups vs. streptozotocin alone group.

^d^denotes significance of the adenine + streptozotocin + flaxseed treated group vs. adenine + streptozotocin group.

### Biochemical and urinary parameters

Table 3 shows the effect of adenine, STZ and flaxseed alone, and in different combinations on various biochemical parameters. Adenine and flaxseed did not exert any significant impact on blood sugar levels when given alone. STZ, on the other hand, given alone or concomitantly with adenine, caused a significant rise in blood sugar levels. Flaxseed significantly lowered the elevation induced by streptozotocin in the blood glucose levels.

**Table 3:** Effect of flaxseed (FLX) treatment on some plasma parameters in control rats (CON), and rats administered concomitantly adenine (A) and /or streptozotocin (STZ).

Parameters/ Treatment	CON	ADE	STZ	ADE+STZ	FLX	ADE+FLX	STZ+FLX	ADE/STZ/FLX
Glucose (mmol/L)	6.21±0.37	4.29±0.39	23.28±1.16^**a**^	20.19±1.00^**a**^^**b**^^**c**^	5.64±0.31	4.82±0.36	15.55±1.06^**a**^^**c**^	14.29±0.44^**a**^^**b**^^**c**^^**d**^
Creatinine (μmol/L)	17.45±1.04	57.82±3.84^**a**^	31.30±2.23^**a**^	65.42±4.23^**a**^^**b**^^**c**^	16.73±0.85	36.83±2.78^**a**^^**b**^	25.12±1.43^**a**^	48.38±2.38^**a**^^**b**^^**c**^^**d**^
Urea (mmol/L)	3.73±0.25	16.41±0.99^**a**^	6.98±0.55^**a**^	19.28±0.89^**a**^^**b**^^**c**^	3.68±0.24	9.57±0.38^**a**^^**b**^	5.13±0.35	14.57±1.09^**a**^^**c**^^**d**^
Uric acid (μmol/L)	29.03±1.98	71.38±4.93^**a**^	57.47±4.55^**a**^	93.22±7.47^**a**^^**b**^^**c**^	26.48±2.60	44.78±3.19^**a**^^**b**^	42.43±3.52^**a**^^**c**^	79.87±6.15^**a**^^**c**^^**d**^
Calcium (mmol/L)	0.69±0.05	0.37±0.02^**a**^	0.63±0.04	0.35±0.04^**a**^^**c**^	0.72±0.04	0.49±0.02^**a**^^**b**^	0.70±0.03	0.47±0.02^**a**^^**b**^^**c**^^**d**^
Phosphorus (mmol/L)	0.79±0.04	1.25±0.07^**a**^	1.01±0.06^**a**^	1.48±0.08^**a**^^**b**^^**c**^	0.83±0.03	1.00±0.09^**a**^^**b**^	0.89±0.05	1.26±0.07^**a**^^**c**^^**d**^

Values in the table are means ± SEM (n = 6).

Diabetes was induced by a single intraperitoneal injection of streptozotocin (55 mg/kg) and chronic kidney disease by adenine (150 mg/kg/day) by oral gavage and flaxseed (15% w//w) was given throughout the experiment by oral gavage.

Differences between the groups were assessed by one-way analysis of variance (ANOVA) followed by Bonferroni’s multiple comparison test, where P < 0.05.

^a^denotes significance of different groups vs. control group.

^b^denotes significance of different adenine treated groups vs. adenine alone group.

^c^denotes significance of different streptozotocin groups vs. streptozotocin alone group.

^d^denotes significance of the adenine + streptozotocin + flaxseed treated group vs. adenine + streptozotocin group.

Adenine and STZ, given singly or in combination, induced a significant increase in plasma creatinine, urea, uric acid and phosphorus when compared with control. Flaxseed significantly abated the change caused by adenine but not STZ except for uric acid. However, it significantly lessened the changes induced by concomitant administration of adenine and STZ.

Adenine, STZ or their combination caused a significant decrease in creatinine clearance, urinary creatinine and urine osmolality when compared with the control. They also caused a significant increase in NAG level, the ratio of urinary albumin/creatinine and the urinary NAG/creatinine ratio. In addition, STZ significantly increased urinary excretion of glucose. The addition of flaxseed to adenine, STZ or their combination significantly mitigated these changes except for the effect of STZ and its concomitant administration with adenine on urinary creatinine and NAG (Table 4).

**Table 4:** Effect of flaxseed (FLX) treatment on some physiological urinary parameters in control (CON) rats, and rats administered concomitantly adenine (A) and /or streptozotocin (STZ).

Parameters/ Treatment	CON	ADE	STZ	ADE+STZ	FLX	ADE+FLX	STZ+FLX	ADE/STZ/FLX
Creatinine clearance (mL/min)	2.10±0.12	0.35±0.02^**a**^	0.64±0.04^**a**^	0.29±0.02^**a**^^**c**^	2.57±0.17^**a**^	1.25±0.10^**a**^^**b**^	1.69±0.03^**a**^^**c**^	0.63±0.05^**a**^^**b**^^**d**^
Creatinine (mg/dL)	54.40±1.45	8.80±0.49^**a**^	2.51±0.17^**a**^	2.17±0.10^**a**^^**b**^	56.82±3.96	26.32±1.88^**a**^^**b**^	7.75±0.25^**a**^^**c**^	5.06±0.17^**a**^
Osmolality (Osmol/Kg)	2.02±0.08	0.53±0.03^**a**^	0.50±0.02^**a**^	0.43±0.03^**a**^	1.95±0.10	1.10±0.07^**a**^^**b**^	0.74±0.05^**a**^^**c**^	0.69±0.05^**a**^^**c**^^**d**^
Glucose excretion (mmol/24 h)	0.01±0.00	0.01±0.00	4.28±0.16^**a**^	4.85±0.16^**a**^^**b**^^**c**^	0.01±0.00	0.01±0.00	3.20±0.19^**a**^^**c**^	3.95±0.18^**a**^^**b**^^**d**^
UACR (μg/mmol)	0.51±0.03	1.44±0.08^**a**^	3.17±0.20^**a**^	5.16±0.32^**a**^^**b**^^**c**^	0.42±0.03	0.70±0.04^**b**^	1.12±0.04^**a**^^**c**^	2.11±0.16^**a**^^**b**^^**c**^^**d**^
NAG (IU/L)	4.78±0.34	8.17±0.40^**a**^	7.06±0.36^**a**^	10.00±0.73^**a**^^**b**^^**c**^	4.59±0.42	6.32±0.58^**a**^^**b**^	6.19±0.28^**a**^	8.81±0.55^**a**^^**c**^
UNCR (IU/mmol)	0.99±0.06	10.58±0.57^**a**^	32.02±0.95^**a**^	51.82±2.10^**a**^^**b**^^**c**^	0.91±0.06	2.75±0.24^**b**^	9.05±0.41^**a**^^**c**^	19.68±1.01^**a**^^**b**^^**c**^^**d**^

Values in the table are means ± SEM (n = 6).

Diabetes was induced by a single intraperitoneal injection of streptozotocin (55 mg/kg) and chronic kidney disease by adenine (150 mg/kg/day) by oral gavage and flaxseed (15% w//w) was given throughout the experiment by oral gavage. NAG: N-Acetyl-β-D-Glucosaminidase, UACR: urinary albumin creatinine ratio, UNCR: urinary N-Acetyl-β-D-Glucosaminidase creatinine ratio.

Differences between the groups were assessed by one-way analysis of variance (ANOVA) followed by Bonferroni’s multiple comparison test, where P < 0.05.

^a^denotes significance of different groups vs. control group.

^b^denotes significance of different adenine treated groups vs. adenine alone group.

^c^denotes significance of different streptozotocin groups vs. streptozotocin alone group.

^d^denotes significance of the adenine + streptozotocin + flaxseed treated group vs. adenine + streptozotocin group.

Table 5 illustrates the effects of adenine, STZ and flaxseed alone and different combinations on some measured plasma biomarkers of kidney injury. Both adenine and STZ or their combination significantly elevated the indoxyl sulfate concentrations, cystatin C, NGAL and AGEs, and significantly decreased the concentration of renalase. Flaxseed was able to significantly mitigate all these induced changes except for the effect of concomitant treatment of adenine and STZ on renalase concentration.

**Table 5:** Effect of flaxseed (FLX) treatment on indoxyl sulfate, cystatin C, renalase and neutrophil gelatinase-associated lipocalin (NGAL) in control (CON) rats, and rats administered concomitantly adenine (A) and /or streptozotocin (STZ).

Parameters/ Treatment	CON	ADE	STZ	ADE+STZ	FLX	ADE+FLX	STZ+FLX	ADE/STZ/FLX
Indoxyl sulfate (μg/mL)	3.07±0.20	28.28±1.06^**a**^	15.54±1.11^**a**^	34.83±2.51^**a**^^**b**^^**c**^	3.72±0.34	16.35±0.91^**a**^^**b**^	10.81±0.90^**a**^^**c**^	25.09±2.07^**a**^^**c**^^**d**^
Cystatin C (ng/mL)	12.32±0.60	29.94±2.35^**a**^	20.32±0.84^**a**^	22.66±2.66^**a**^^**b**^	9.41±1.42	14.81±2.25^**b**^	11.38±1.30^**c**^	15.02±1.75^**b**^^**c**^^**d**^
Renalase (μg/mL)	9.62±0.52	4.22±0.25^**a**^	3.55±0.26^**a**^	2.86±0.23^**a**^^**b**^	9.96±0.66	6.08±0.39^**a**^^**b**^	4.97±0.36^**a**^^**c**^	3.91±0.19^**a**^
NGAL (ng/mL)	24.04±1.82	69.71±1.46^**a**^	45.41±1.99^**a**^	68.94±1.27^**a**^^**c**^	21.31±1.68	42.16±6.51^**a**^^**b**^	26.74±1.72^**c**^	43.21±5.08^**a**^^**b**^^**d**^
AGEs	0.15±0.01	0.35±0.02^**a**^	0.31±0.02^**a**^	0.40±0.03^**a**^^**c**^	0.12±0.01	0.21±0.02^**a**^^**b**^	0.23±0.02^**a**^^**c**^	0.26±0.02^**a**^^**b**^

Values in the table are means ± SEM (n = 6).

Diabetes was induced by a single intraperitoneal injection of streptozotocin (55 mg/kg) and chronic kidney disease by adenine (150 mg/kg/day) by oral gavage and flaxseed (15% w//w) was given throughout the experiment by oral gavage. NAGL: neutrophil gelatinase-associated lipocalin; AGEs: advanced glycation end products.

Differences between the groups were assessed by one-way analysis of variance (ANOVA) followed by Bonferroni’s multiple comparison test, where P < 0.05.

^a^denotes significance of different groups vs. control group.

^b^denotes significance of different adenine treated groups vs. adenine alone group.

^c^denotes significance of different streptozotocin groups vs. streptozotocin alone group.

^d^denotes significance of the adenine + streptozotocin + flaxseed treated group vs. adenine + streptozotocin group.

### Oxidative and inflammatory indices

**Fig 1:**
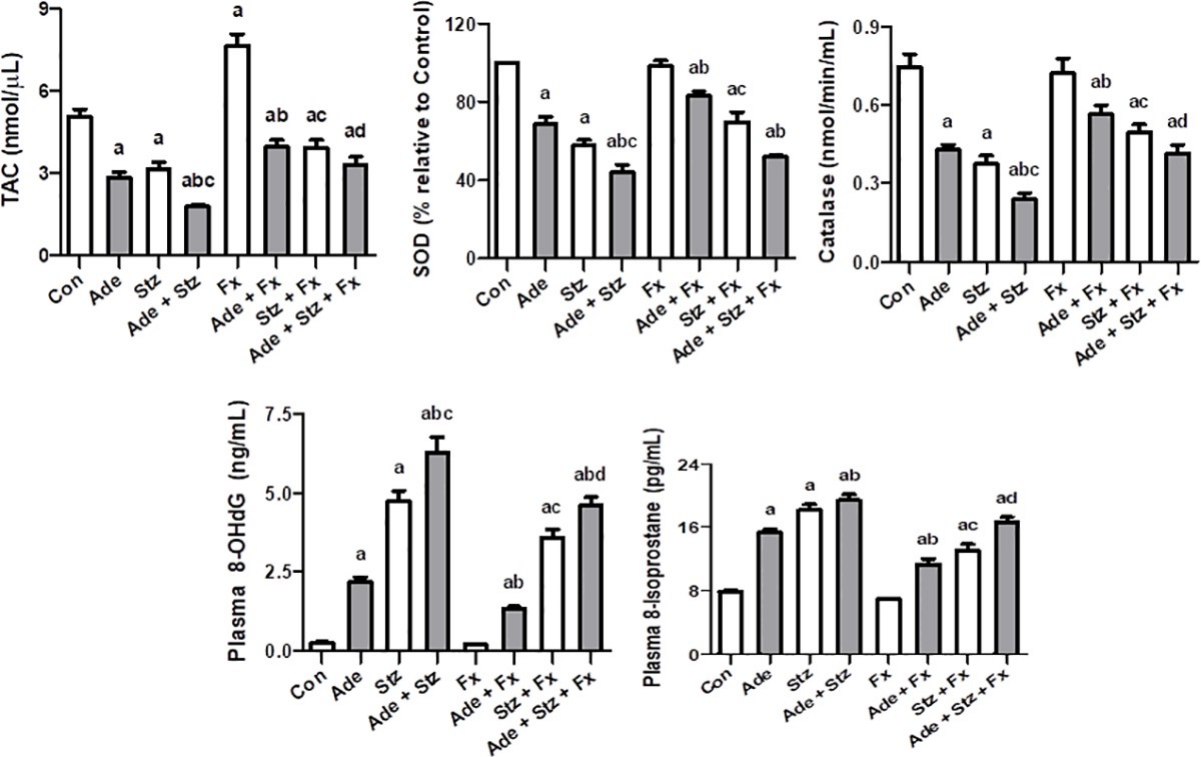
Effect of flaxseed treatment (Fx, 15% w/w) on the renal concentrations of total antioxidant capacity (TAC), superoxide dismutase (SOD), catalase, 8-hydroxy-2’-deoxyguanosine (8-OHdG) and 8-isoprostane in control (Con) rats, and rats administered adenine (Ade, 150 mg/Kg/day), streptozotocin (Stz, 55 mg/Kg) or a combination of these. Each vertical column with bar represents the mean ± SEM (n = 6). Differences between the groups were assessed by one-way analysis of variance (ANOVA) followed by Bonferroni’s multiple comparison test, where P < 0.05. ^a^denotes significance of different groups vs. control group. ^b^denotes significance of different adenine treated groups vs. adenine alone group. ^c^denotes significance of different streptozotocin groups vs. streptozotocin alone group. ^d^denotes significance of the adenine + streptozotocin + flaxseed treated group vs. adenine + streptozotocin group.

The effect of the different treatments on some of the antioxidant biomarkers is illustrated in Fig 1. Adenine and STZ or their combination significantly decreased TAC, SOD, and catalase levels, and significantly increased the levels of 8-OHdG and 8-isoprostane. Flaxseed significantly abated these changes except for the effect of concomitant use of adenine and STZ on SOD.

**Fig 2:**
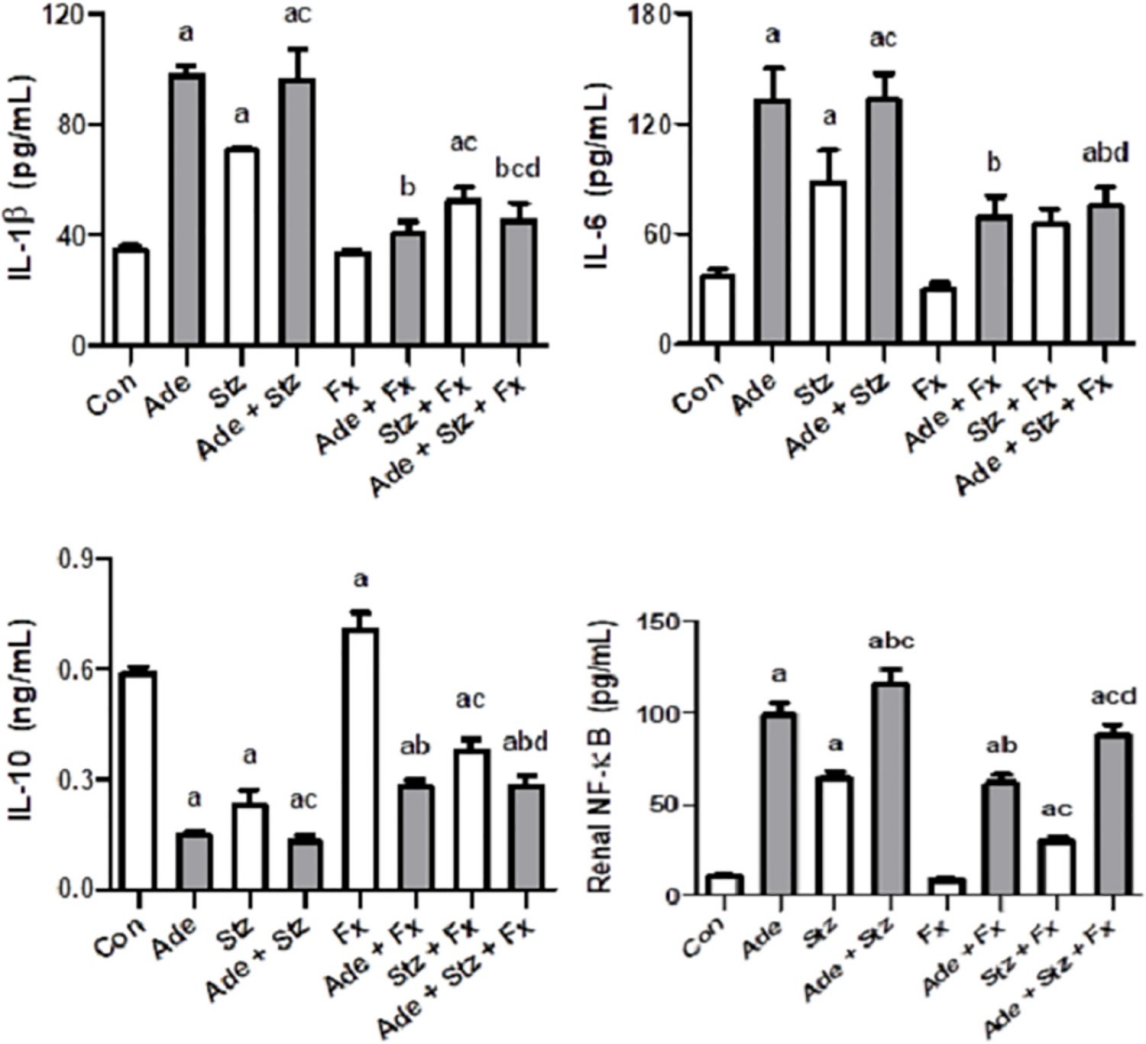
Effect of flaxseed treatment (Fx, 15% w/w) on the renal concentrations of interleukin-1β (IL-1β), interleukin-6 (IL-6), interleukin-10 (IL-10) and nuclear factor kappa-light-chain-enhancer of activated B cells (NF-κB) in control (Con) rats, and rats administered adenine (Ade, 150 mg/Kg/day), streptozotocin (Stz, 55 mg/Kg) or a combination of these. Each vertical column with bar represents the mean ± SEM (n = 6). Differences between the groups were assessed by one-way analysis of variance (ANOVA) followed by Bonferroni’s multiple comparison test, where P < 0.05. ^a^denotes significance of different groups vs. control group. ^b^denotes significance of different adenine treated groups vs. adenine alone group. ^c^denotes significance of different streptozotocin groups vs. streptozotocin alone group. ^d^denotes significance of the adenine + streptozotocin + flaxseed treated group vs. adenine + streptozotocin group.

Fig 2 depicts the effect of various treatments on some of the measured anti-inflammatory biomarkers. Adenine and STZ or their combination significantly raised IL-1 β, IL-6 and NF-κB and decreased the levels of IL-10. Flaxseed significantly mitigated these changes.

### Histopathological changes

**Fig 3:**
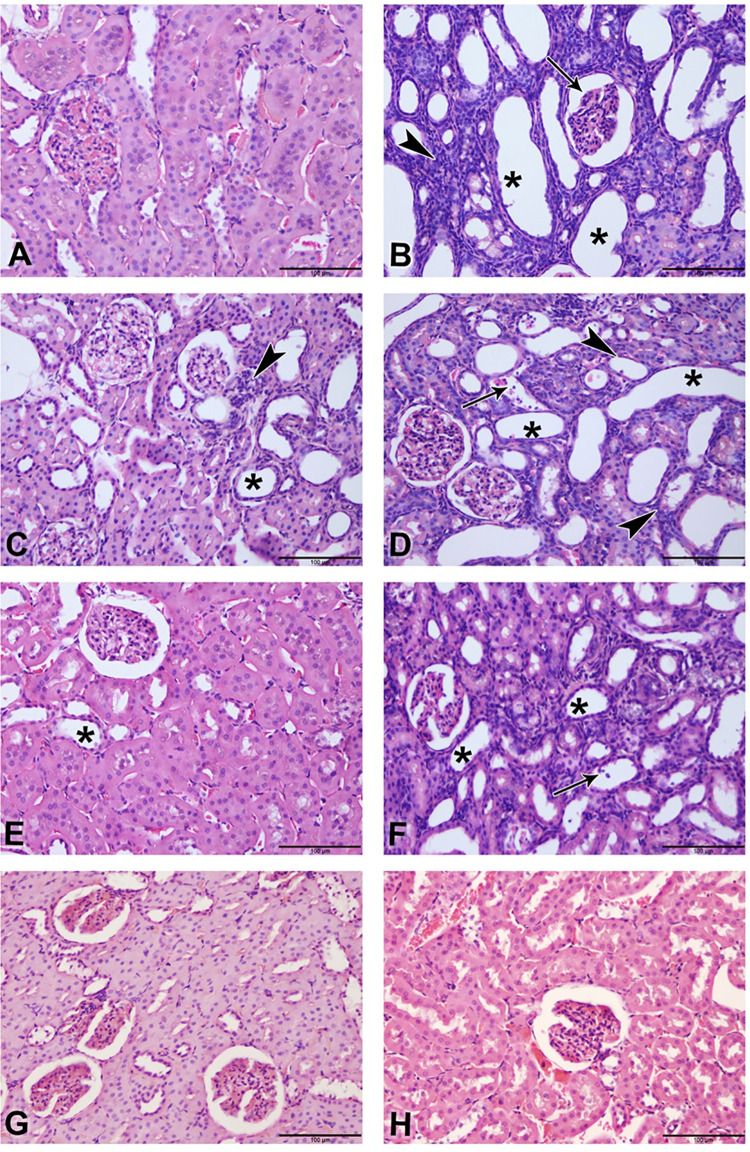
Photomicrograph of renal tissues (Bar = 100μm, H&E stain): (**A**; control group) showing normal renal tissue with intact glomeruli and renal tubules (Score 0); (**B**; adenine group) showing marked renal tubular necrosis with pyknotic nuclei (arrowheads), cystic dilatation of multiple renal tubules (asterisks), marked basophilia, and dilatation of bowman’s capsule (arrow) (Score 4); (**C**; streptozotocin group) showing moderate basophilia with cystic dilation of few renal tubules (asterisk) and focal aggregates of mononuclear cells (arrowhead) (Score 2); (**D**; adenine + streptozotocin group) showing marked basophilia, renal tubular necrosis (arrowheads), cystic dilatation of multiple renal tubules (asterisks), and cellular casts in multiple tubules (arrow) (Score 4); (**E**; flaxseed group) showing histologically normal renal tubules with the exception of few dilated tubules (asterisk) (Score 1); (**F**; adenine + flaxseed group) showing tubular basophilia, tubular dilatations (asterisks) and cellular casts (arrow) (Score 3); (**G**; streptozotocin + flaxseed group) showing normal histological structures of the majority of the renal tubules with intact glomeruli (Score 1); (**H**; adenine + streptozotocin + flaxseed group) showing normal histological structures of the renal tubules with intact glomeruli (Score 0).

**Fig 4:**
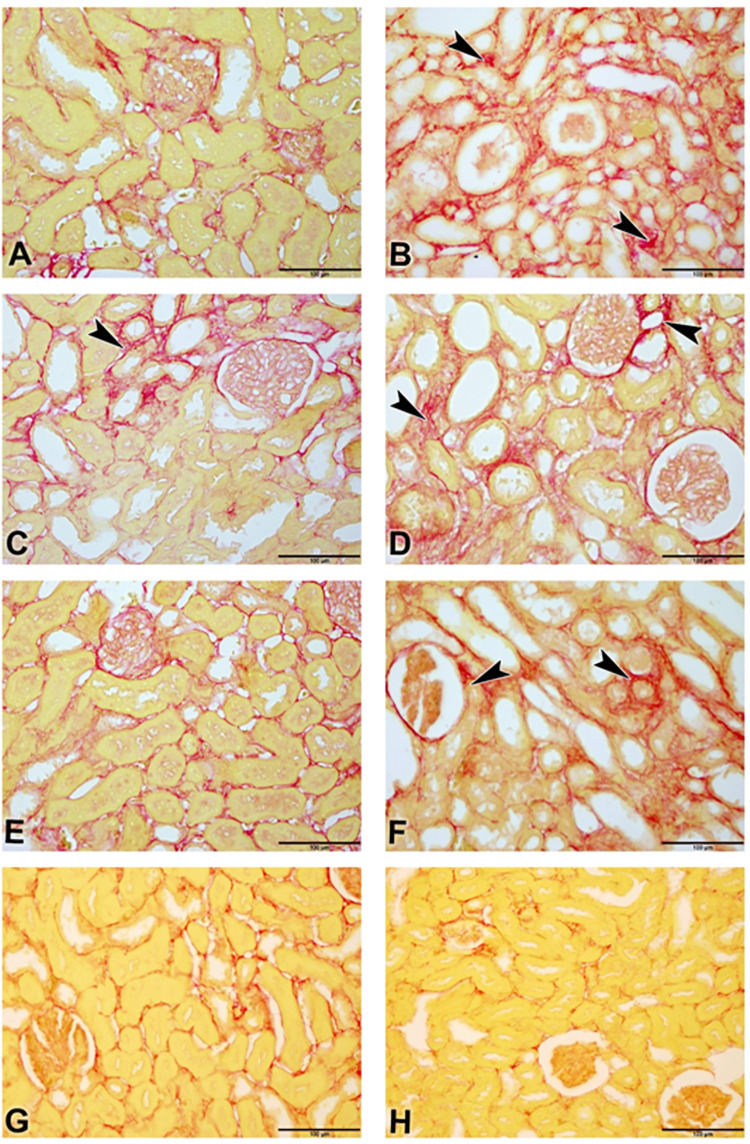
Photomicrograph of renal tissues (Bar = 100μm, A-G; Picro-Sirius red); renal tissues of groups A-H (**A**: control, **B**: adenine, **C**: streptozotocin, **D**: adenine + streptozotocin, **E**: flaxseed, **F**: adenine + flaxseed, **G**: streptozotocin + flaxseed, **H**: adenine + streptozotocin + flaxseed) showing the distribution of Sirius Red-stained fibrotic areas fibers (arrowheads) and yellow non-collagen structures.

**Fig 5:**
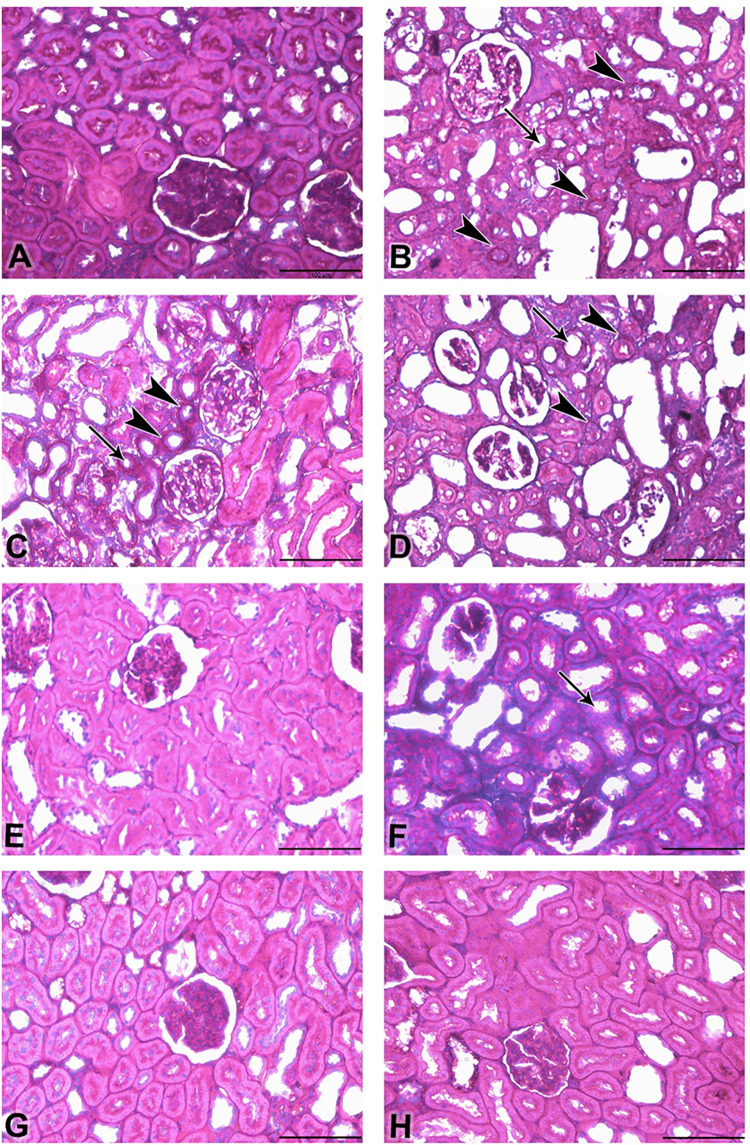
Photomicrograph of renal tissues (Bar = 100μm, A-G; Periodic Acid Schiff (PAS)); renal tissues of groups A-H (**A**: control, **B**: adenine, **C**: streptozotocin, **D**: adenine + streptozotocin, **E**: flaxseed, **F**: adenine + flaxseed, **G**: streptozotocin + flaxseed, **H**: adenine + streptozotocin + flaxseed), showing tubular atrophy represented by a thickened tubular basement membrane (arrowheads) stained positive (magenta red) for PAS and loss of tubular brush borders (arrows).

The photomicrographs of renal tissues for H & E stain, Sirius red stain and Picro-Sirius red stain are depicted in Figs 3 and 4, respectively. Table 6 shows the lesions score and fibrosis index. Control (3A) showed normal renal tissue with intact glomeruli and renal tubules (Score 0), Adenine group (3B) showed marked renal tubular necrosis with pyknotic nuclei (arrowheads), cystic dilatation of multiple renal tubules (asterisks), marked basophilia, and dilatation of Bowman’s capsule (arrow) (Score 4). STZ (3C) showed moderate basophilia with cystic dilation of few renal tubules (asterisk) and focal aggregates of mononuclear cells (arrowhead) (Score 2). Adenine + STZ group (3D) showed marked basophilia, renal tubular necrosis (arrowheads), cystic dilatation of multiple renal tubules (asterisks), and cellular casts in multiple tubules (arrow) (Score 4). Flaxseed alone group (3E) showed histologically normal renal tubules except for few dilated tubules (asterisk) (Score 1). Adenine + Flaxseed group (3F) showed tubular basophilia, tubular dilatations (asterisks) and cellular casts (arrow) (Score 3) while STZ + Flaxseed group (3G) showed normal histological structures of the majority of the renal tubules with intact glomeruli (Score 1). Adenine + STZ + Flaxseed group (3H) showed normal histological structures of the renal tubules with intact glomeruli (Score 0). Fig 4 shows the distribution of Sirius Red-stained fibrotic areas fibers (arrowheads) and yellow non-collagen structures in various groups. Fig 5 illustrates the Periodic Acid Schiff (PAS) staining where the tubular atrophy represented by a thickened tubular basement membrane (arrowheads) and stained positive (magenta red) for PAS and loss of tubular brush borders (arrows) are shown.

**Table 6:** Effect of treatment with flaxseed (FLX) on histopathological assessment of kidney sections in control (CON) rats, and rats treated concomitantly with adenine (A) and /or streptozotocin (STZ).

Parameters/ Treatment	CON	ADE	STZ	ADE+STZ	FLX	ADE+FLX	STZ+FLX	ADE/STZ/FLX
Lesion score (tubular necrosis)	0	4	2	4	1	3	1	0
Fibrosis index (%)	4.8	32.7	15.4	28.8	8.5	22.4	7.2	5.2

Diabetes was induced by a single intraperitoneal injection of streptozotocin (55 mg/kg) and chronic kidney disease by adenine (150 mg/kg/day) by oral gavage and flaxseed (15% w//w) was given throughout the experiment by oral gavage.

## Discussion

DM is one of the most common non-communicable diseases in the world. It can lead to multiple complications, and is associated with various comorbidities that can substantially dimmish the quality of life of affected patients [7, 27]. CKD is one of those complications that carries high morbidity and mortality [4]. In this study, we explored the impact of dietary flaxseed on CKD in DM using an animal model as a translational bridge for possible future clinical trials in humans, pending further pharmacological and toxicological investigations.

DM in this study was experimentally induced by intraperitoneal injection of STZ, while CKD was induced by the administration of adenine via oral gavage. Both conditions were evident by the physiological, biochemical, and histological results reported in this study and other studies [28–31]. STZ caused a significant rise in blood glucose and caused derangement in plasma and urinary creatinine, urea, albumin and NAG activity. Adenine caused typical physiological changes in the weight, water and food intake and urine flow reported previously with CKD [30, 31]. It also significantly altered the urinary and plasma concentrations of urea, creatinine, albumin and NAG activity. Both STZ and adenine significantly caused elevations in biochemical markers (indoxyl sulfate, cystatin C and NGAL activity), inflammatory indices (IL-1 β, IL-6, IL-10 and NF-κB) and decreased antioxidant capacities (TAC, SOD and catalase activities).

Flaxseed, similar to other natural compounds such as curcumin, sesamin and chrysin, contains several components that have anti-inflammatory, antioxidant and antiapoptotic actions [32–36]. It contains fiber, lignans, polyunsaturated fatty acids and other bioactive compounds which collectively can decrease inflammation, oxidative stress and improves hemodynamic status [16, 37]. For example, the consumption of flaxseed was found to have cardioprotective effects via lowering the blood pressure, the circulating lipids and by exerting antiplatelet and antiatherosclerotic actions [38].

Flaxseed has been shown to play a protective role against oxidative stress, which is evident by the increase in antioxidants enzymes such as CAT, SOD and glutathione peroxidase [21]. Oxidative stress can cause tissue damage by generating the reactive oxygen species and leading to chronic inflammation [39]. Oxidative stress also increases DNA oxidation, lipid peroxidation and the depletion of antioxidant enzymes, promoting further tissue injury and ischemia [40]. Flaxseed in this study enhanced the TAC, SOD, and catalase activities, possibly through the upregulation of the genes expressing these enzymes. The increase in these enzyme activities might have led to a reduction in the direct oxidative and peroxidative markers 8-OHdG and 8-isoprostane, respectively. It is also possible that flaxseed downregulated the genes expressing these markers [41]. In addition, the modulation of oxidative stress by flaxseed was reflected indirectly in the improvement in the creatine clearance and the concentration of other biochemical markers such as indoxyl sulfate, NAG, renalase, cystatin C and NGAL.

Flaxseed was also reported to play a role in the inflammatory and apoptosis processes. It can decrease the pro-inflammatory cytokines such as IL-6, IL-1, TNF-α and NF-κB and promotes a pro-apoptotic and anti-angiogenic effect in tumours such as ovarian tumours [37, 42–44]. Adenine and STZ have been shown to trigger inflammation and apoptosis in renal tissues leading to a nephropathic status [28–31]. This is demonstrated by the significant increase in the pro-inflammatory cytokines IL-Iβ, IL-6 and NF-κB. The addition of flaxseed to STZ and/or adenine significantly reduced the expression of these pro-inflammatory cytokines. This effect might be attributed to the flaxseed component, α-linolenic acid, which was shown to reduce the production of pro-inflammatory cytokines via the downregulation of their gene expression [45]. It has also been reported previously that inhibition of NF-κB activation can downregulate the inflammatory response [46]. Furthermore, the coadministration of flaxseed here increased the concentration of IL-10. The cytokine IL-10, also known as human cytokine synthesis inhibitory factor, and expressed by many immune cells, is regarded as a regulatory cytokine that inhibits innate and adaptive inflammatory responses that help protect tissue from the damage that might be caused by exacerbated adaptive immunity [47, 48].

At the histopathological level, flaxseed reduced both the tubular necrosis and fibrosis induced by adenine and/or STZ. Such findings at the tissue level confirm the beneficial role of flaxseed in reducing the inflammatory and oxidative reactions associated with diabetes and CKD.

The effects shown in the current experimental study verified that flaxseed effectively mitigated nearly all the physiological, biochemical, and histopathological alterations caused by adenine and/or STZ which suggest that it can be considered a potentially useful dietary supplement in the management of diabetic induced-CKD. The antioxidant and anti-inflammatory properties of flaxseed have been used to mitigate inflammatory and oxidative cascades in some human diseases. For example, flaxseed supplementation modulated the inflammatory and oxidative stress biomarkers in cystic fibrosis and ulcerative colitis patients [49, 50]. In addition, flaxseed was found to be effective in ameliorating some of the symptoms of metabolic syndrome and decreasing blood pressure and lipid peroxidation [51].

In conclusion, the obtained results suggest that flaxseed can be a potential therapeutic agent for the treatment of diabetes-induced CKD. Further studies either at the translational level or small-scale clinical trials might be warranted to confirm such findings.

## Supporting information

S1 File Data for Fig 1. (XLSX)

S2 File Date for Fig 2. (XLSX)

S3 File Data for Table 2. (XLSX)

S4 File Date for Table 3. (XLSX)

S5 File Data for Table 4. (XLSX)

S6 File Date for Table 5. (XLSX)
